# High-Fat Diet Modulates the Excitability of Neurons within the Brain–Liver Pathway

**DOI:** 10.3390/cells12081194

**Published:** 2023-04-20

**Authors:** Adrien J. R. Molinas, Lucie D. Desmoulins, Roslyn K. Davis, Hong Gao, Ryousuke Satou, Andrei V. Derbenev, Andrea Zsombok

**Affiliations:** 1 Department of Physiology, School of Medicine, Tulane University, New Orleans, LA 70130, USA; amolinas@tulane.edu (A.J.R.M.); ldesmoulins@tulane.edu (L.D.D.); rdavis31@tulane.edu (R.K.D.); rsato@tulane.edu (R.S.); aderben@tulane.edu (A.V.D.); 2 Tulane Brain Institute, Tulane University, New Orleans, LA 70130, USA

**Keywords:** paraventricular nucleus, ventral brainstem, sympathetic nervous system, pseudorabies virus, liver, high-fat diet, patch-clamp electrophysiology, brain–liver pathway

## Abstract

Stimulation of hepatic sympathetic nerves increases glucose production and glycogenolysis. Activity of pre-sympathetic neurons in the paraventricular nucleus (PVN) of the hypothalamus and in the ventrolateral and ventromedial medulla (VLM/VMM) largely influence the sympathetic output. Increased activity of the sympathetic nervous system (SNS) plays a role in the development and progression of metabolic diseases; however, despite the importance of the central circuits, the excitability of pre-sympathetic liver-related neurons remains to be determined. Here, we tested the hypothesis that the activity of liver-related neurons in the PVN and VLM/VMM is altered in diet-induced obese mice, as well as their response to insulin. Patch-clamp recordings were conducted from liver-related PVN neurons, VLM-projecting PVN neurons, and pre-sympathetic liver-related neurons in the ventral brainstem. Our data demonstrate that the excitability of liver-related PVN neurons increased in high-fat diet (HFD)-fed mice compared to mice fed with control diet. Insulin receptor expression was detected in a population of liver-related neurons, and insulin suppressed the firing activity of liver-related PVN and pre-sympathetic VLM/VMM neurons in HFD mice; however, it did not affect VLM-projecting liver-related PVN neurons. These findings further suggest that HFD alters the excitability of pre-autonomic neurons as well as their response to insulin.

## 1. Introduction

The liver, a central organ in carbohydrate and lipid metabolism, is regulated by both branches of the autonomic nervous system (ANS). In general, increased activity of the sympathetic nervous system (SNS) leads to elevation of hepatic glucose production, whereas increased activity of the parasympathetic nervous system decreases glucose production [1]. In type 2 diabetes mellitus, excessive hepatic glucose production, which includes increased glycogenolysis and elevated gluconeogenesis, is one of the major contributors to hyperglycemia [2]. Increased sympathetic activity is recognized in obese and diabetic conditions and plays a significant role in the development and/or progression of diseases associated with dysregulated glucose homeostasis [3,4]. Mice fed with a high-fat diet (HFD) develop obesity, hyperinsulinemia, and hyperglycemia and exhibit elevation of hepatic glucose production, demonstrated by increased expression of phosphoenolpyruvate carboxykinase (PEPCK) and glucose-6-phosphatase (G6Pase) and by hyperinsulinemic-euglycemic clamp studies [5,6]. Intriguingly, increased sympathetic activity was also observed in diet-induced obese mice [7], which likely contributes to increased hepatic glucose production.

Many hypothalamic nuclei, including the paraventricular nucleus (PVN) of the hypothalamus, contribute to the regulation of autonomic outflow and modulate whole-body metabolism [7,8,9]. The contribution of the PVN to the regulation of glucose homeostasis is well accepted [8,9,10,11]. Stimulation of PVN neurons results in hyperglycemia, which was absent in rats with sympathectomy, indicating that the hyperglycemia was due to the PVN-originated activation of the SNS [12]. The activity of pre-autonomic neurons, neurons with direct or indirect projections to pre-ganglionic neurons, largely influences the activity of the sympathetic and parasympathetic outflow. In the case of the SNS, the information from pre-sympathetic PVN neurons is transmitted through either direct connections to the pre-ganglionic neurons in the spinal cord and/or through pre-sympathetic neurons, located mainly in the ventrolateral medulla (VLM), which includes the rostral ventrolateral medulla (RVLM), and in the ventromedial medulla (VMM), which includes the gigantocellular reticular nucleus and the raphe pallidus with projections to spinal pre-ganglionic neurons [13,14,15].

In previous studies, pseudorabies virus (PRV), a retrograde, trans-synaptic viral tracer, was used to identify liver-related neurons in the central nervous system [16,17,18]. Electrophysiological recordings established the synaptic regulation of liver-related PVN neurons in control and hyperglycemic mice [19] and also demonstrated that liver-related PVN neurons are more active in *db*/*db* mice compared to lean mice [20]. Intriguingly, the cellular properties of liver-related pre-sympathetic neurons remain to be determined. Here, we tested the hypotheses that a high-fat diet increases the excitability of liver-related PVN neurons and alters the response to insulin in pre-sympathetic liver-related neurons. Liver-related neurons were identified in the PVN and ventral brainstem with PRV, and patch-clamp recordings were conducted from liver-related PVN neurons, ventral brainstem-projecting PVN neurons, and pre-sympathetic liver-related neurons in the VLM/VMM. We found that the excitability of liver-related PVN neurons increased in HFD-fed mice compared to mice fed with the control diet, and in HFD mice, insulin decreased the excitability of liver-related PVN neurons. Similarly, insulin decreased the activity of pre-sympathetic liver-related neurons in the ventral brainstem in HFD-fed mice; however, it did not have a significant effect on liver-related PVN neurons projecting to the VLM. Together, this is the first study to determine the effect of HFD and insulin on neurons involved in the regulation of liver functions, with a particular focus on pre-sympathetic neurons. Indeed, our study shows that liver-related neurons are heterogeneous, and only specific populations of neurons are altered by HFD and insulin.

## 2. Materials and Methods

### 2.1. Animals

Male C57BL/6J DIO mice (Jackson Lab # 380050, Bar Harbor, ME, USA) and their respective controls (Jackson Lab # 380056) received either a rodent diet 60 kcal% fat (Research Diets D12492, Research Diets Inc., New Brunswick, NJ, USA) or a 10 kcal% fat (Research Diets D12450B) from 6 weeks of age to induce obesity. The mice had free access to their respective diet and water, were kept in a room with 12 h/12 h dark/light cycle, and were sacrificed at 15–19 weeks. Experiments were conducted based on the guidelines of the National Institutes of Health Guide for the Care and Use of Laboratory Animals and approved by Tulane University’s Institutional Animal Care and Use Committee (Protocol 1398).

### 2.2. Pseudorabies Virus Inoculation

Pseudorabies virus 152 (PRV-152; reports enhanced green fluorescence protein [EGFP], supplied by NCRR CNNV Virus Center), a retrogradely transported pseudorabies virus, was used to label liver-related neurons, as described previously [19,21,22]. Under anesthesia, two injections (2 μL per site) were made into the parenchyma of the left lateral lobe. A drop of tissue adhesive (Vetbond, 3M) was used to seal each injection site to prevent the leakage of the virus. The mice were maintained in a biosafety level 2 facility up to 96 h post-inoculation for experiments in the ventral brainstem, and up to 115 h post-inoculation for experiments in the PVN. In some cases, mice received a second viral injection (PRV-614; reports red fluorescence protein (RFP)) into the ventral brainstem to distinguish VLM-projecting liver-related neurons in the PVN.

### 2.3. Brain Injections

In a set of mice, brain injections were performed under isofluorane anesthesia using a stereotaxic apparatus (Stoelting Co., Wood Dale, IL, USA) to facilitate accurate, bilateral viral injections into the VLM (6.5 mm posterior; 1 mm lateral; 6.3 mm ventral to Bregma). The coordinates were chosen according to the Paxinos mouse brain atlas [23]. PRV-614 (200 nL, 1 nL/s) was injected using a glass micropipette connected to a nano-injector system (Nanoject III, Drummond, Sci. Co., Broomall, PA, USA). The micropipette remained in place for 7 min before removal to prevent backflow. The incision site was closed using surgical nylon sutures (Johnson and Johnson, New Brunswick, NJ, USA).

### 2.4. Brain Slices Preparation

Acute brain slices were made as described previously [19,24]. After anesthesia with isoflurane, the mouse was decapitated, and the brain was removed and immersed in ice-cold oxygenated artificial cerebrospinal fluid (aCSF) containing the following (in mM): 124 NaCl, 26 NaHCO_3_, 1.4 NaH_2_PO_4_, 11 D-glucose, 3 KCl, 1.3 MgCl_2_, 1.5 CaCl_2_, pH 7.3–7.4. Coronal hypothalamic slices containing the PVN (300 µm) or brainstem slices containing the VLM/VMM (300 μm) were made using a vibrating microtome. The slices were stored in a holding chamber at 34–36 °C, and then transferred to a recording chamber mounted on a fixed stage under an upright microscope (Nikon FN1).

### 2.5. Whole-Cell Patch-Clamp Recordings

Whole-cell patch-clamp recordings were performed at 34–36 °C from liver-related PVN neurons, liver-related PVN neurons projecting to the VLM, and liver-related neurons in the VLM/VMM under a 40× water-immersion objective (N.A = 0.8). Neurons were identified based on their fluorescence, and infrared differential interference contrast optic (IR-DIC) was used to target specific cells. For whole-cell patch-clamp recordings, electrodes (4–6 MΩ) were filled with a solution containing the following (in mM): 135 K^+^ or Cs^+^ gluconate, 10 HEPES, 5 EGTA, 1 NaCl, 1 MgCl_2_, 1 CaCl_2_, 3 KOH or CsOH, 3 Mg-ATP, 0.1% biocytin or 0.1% Neurobiotin 350, pH 7.3–7.4. Electrophysiological signals were recorded using an Axoclamp 700B amplifier (Molecular Devices, LLC., San Jose, CA, USA) and acquired by pClamp (Molecular Devices). Inhibitory post-synaptic currents (IPSCs) were recorded at 0 mV, and excitatory post-synaptic currents (EPSCs) at −60 mV. Bath application of tetrodotoxin (TTX; 1 μM; Tocris Bioscience, Bio-Techne Corporation, Minneapolis, MN, USA) was used to block action potentials and monitor miniature IPSCs (mIPSCs) and EPSCs (mEPSCs).

The excitability of neurons was determined in current clamp mode. In the case of recordings from PVN neurons, the cells were initially hyperpolarized to −90 mV and then depolarizing current steps were applied (1 s duration) to reveal the frequency of action potentials, as shown previously [20,25,26,27]. The effect of insulin (1 µM, Tocris Bioscience,) was revealed on the firing rate and synaptic currents following a ten-minute perfusion. Synaptic currents and action potentials were analyzed offline using pClamp (Molecular Devices, LLC. San Jose, CA, USA) or MiniAnalysis 6.0.7. (Synaptosoft Inc., Decatur, GA, USA). Amplitude and area detection thresholds were set as three times the RMS noise using MiniAnalysis. After the software-based automatic detection, the events were carefully reviewed and manually selected.

### 2.6. Visualization of the Recorded Cells

After recordings, the brain slices containing the neurons filled with neurobiotin 350 (Vector Laboratories, Inc., Newark, CA, USA) or biocytin (Sigma-Aldrich, St. Louis, MO, USA) were fixed in paraformaldehyde (4%) and stored at 4 °C. Following extensive rinse in phosphate buffered saline (0.01 M PBS), slices with biocytin-filled neurons were incubated for 4 h in a solution containing PBS, AMCA Avidin D (1:200), and TritonX-100 (1%) at room temperature. Rinsed slices were mounted on glass slides and coverslipped with Vectashield mounting medium (Vector Laboratories, Inc., Newark, CA, USA). Images were taken with a confocal microscope (Nikon Ti2).

### 2.7. Gene Expression with Droplet Digital PCR

To determine insulin receptor (*Insr*) gene expression in liver-related neurons, droplet digital PCR (ddPCR) was used on single-cell cytoplasm samples from mice kept on control or HFD, as described previously [28]. Briefly, neurons were patched with glass pipettes filled with K^+^-gluconate solution. Whole-cell configuration was maintained for at least 5 min before the cytoplasm was collected. Then, the electrode was rapidly removed, and the tip of the glass pipette was broken at the bottom of an Eppendorf tube containing 10 µL of extraction buffer (PicoPure, ThermoFisher). The intracellular recording solution mixed with the collected cytoplasm (10 µL) was pushed out of the glass pipette. Samples were inverted twice and spun with a tabletop centrifuge, then incubated for 30 min at 42 °C and stored at −80 °C until processing.

Total RNA from single-cell samples was extracted and purified (10 µL) using PicoPure RNA Isolation Kit (ThermoFisher Sci. Inc., Waltham, MA, USA) before testing with ddPCR. Then, 0.2 ng of RNA from mouse liver tissue was used as a positive control and nuclease-free water was used as a negative, no-template control (NTC). Single cell samples (5 µL) and the positive and negative controls were simultaneously tested for the presence of *Insr* mRNA and beta-actin (b-actin) mRNA to validate cytoplasm extraction effectiveness using, respectively, ddPCR Gene Expression Assay dMmuCPE5117544 and dMmuCPE5195285 primer/probe sets from Bio-Rad. Subsequently, dual channel ddPCR was performed with the QX200 ddPCR system (Bio-Rad). The thermal profile used in the ddPCR reaction consisted of 60 min at 45 °C, 10 min at 95 °C, 40 cycles of 30 s at 95 °C and 1 min at 56 °C, 10 min at 98 °C, and infinite holding at 4 °C.

As single-cell samples showed low levels of mRNA, a preamplification step was added. The remaining purified RNA from b-actin mRNA positive samples (5 µL) was used for qPCR. Using the One-Step RT-ddPCR Advanced Kit (Taqman PCR system; Bio-Rad Laboratories, Hercules, CA, USA) and Bio-Rad’s PrimePCR PreAmp (qMmuCID0018034), Insr cDNA was synthetized and amplified. The thermal profile used in the preamplification consisted of 1 h at 45 °C, 10 min at 95 °C, 25 cycles of 30 s at 95 °C and 1 min at 56 °C, 5 min at 72 °C, 10 min at 98 °C, and infinite holding at 4 °C. Then, 1 µL of diluted PCR products was used for ddPCR, as described above, to detect *Insr* cDNA.

### 2.8. Statistical Analysis

To reveal the effect of diet on the excitability of neurons, action potential frequency was analyzed following depolarizing current steps. To determine difference in slope and Y-intercept/elevation of current–action potential frequency, data were fitted with linear or nonlinear regression before using Graphpad test equivalent to an Analysis of Covariance (ANCOVA). A two-tailed *p* < 0.05 was considered significant. Continuous recordings were conducted, and three-minute periods were analyzed with MiniAnalysis (Synaptosoft) to measure peak amplitude and frequency of EPSCs, IPSCs, and action potentials before and after insulin application. Clampfit (Molecular Devices, LLC., San Jose, CA, USA) was used to measure resting membrane potential. Numbers are reported as mean ± standard error (SEM). Statistical analysis was performed using Graphpad Prism 9 software (Graphpad Software, Boston, MA, USA). Outliers were identified and removed using ROUT method (Q = 1%). Kolmogorov–Smirnov test with Dallal–Wilkinson–Lillie for *p*-value was used to test normality of values distribution. Paired and unpaired *t*-tests were used to compare two groups with Gaussian distributed values, while non-Gaussian values were tested with a Mann–Whitney test or a Wilcoxon test.

## 3. Results

### 3.1. Increased Excitability of Liver-Related PVN Neurons in HFD-Fed Mice

To determine the neuronal plasticity of liver-related PVN neurons, patch-clamp recordings were conducted from mice kept on control and high-fat diet (9–13 weeks). As expected, HFD-fed mice were heavier than mice on control diet (HFD: 36.45 ± 0.57 g vs. control: 28.85 ± 0.48 g, *n* = 50 and 48) (unpaired *t*-test, *p* < 0.0001), whereas there was no difference in non-fasting glucose levels measured before sacrifice (HFD: 236.6 ± 16.19 mg/dL vs. control: 227.3 ± 14.64 mg/dL, *n* = 37 and 32) (Mann–Whitney test, *p* = 0.8977).

We found that at baseline, the resting membrane potential and the frequency of action potentials of liver-related PVN neurons were similar in control and HFD-fed mice (unpaired *t*-test and Mann–Whitney test, *p* > 0.05) (Supplementary Figure S1(A1,A2)). In control mice, the average resting membrane potential of liver-related PVN neurons was −45.08 ± 1.49 mV (ranged from −59.00 to −30.70 mV; *n* = 23 from 13 mice) and the majority of the recorded neurons fired spontaneously, with an average frequency of 1.30 ± 0.31 Hz (ranged from 0.01 to 3.75 Hz; *n* = 15 from 9 mice). In HFD-fed mice, the resting membrane potential was −47.91 ± 1.14 mV (ranged from −62.56 to −36.65 mV, *n* = 26 from 14 mice), and most of the recorded neurons had spontaneous firing activity (0.92 ± 0.23 Hz, ranged from 0.01 to 3.22 Hz, *n* = 18 from 12 mice) (Supplementary Figure S1(A1,A2)).

**Figure 1:**
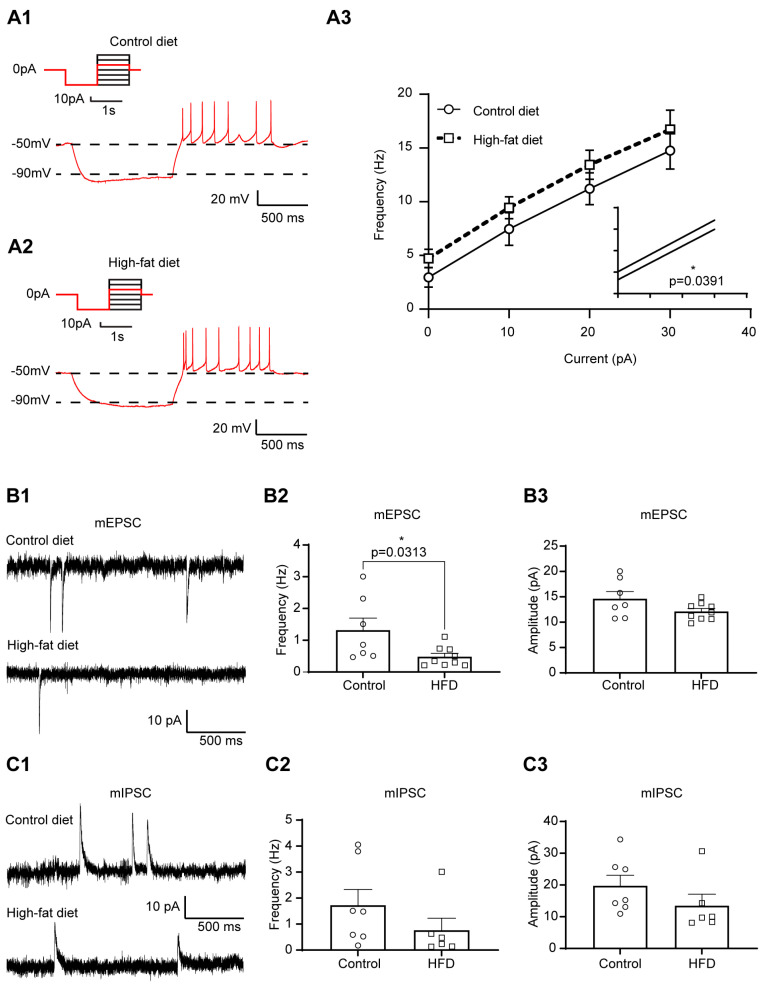
The excitability of liver-related PVN neurons increased in mice kept on HFD. (**A1**,**A2**): Representative traces illustrate the firing rate of liver-related PVN neurons. The recorded neurons were initially hyperpolarized to ~−90 mV, then depolarizing current steps were applied to reveal the firing activity in control (**A1**) and HFD-fed (**A2**) mice. Whereas multiple current steps were applied, only one trace (+10 pA) is shown for better visibility. (**A3**): Current–action potential frequency curves and computed simple linear regression demonstrated that liver-related PVN neurons fire more in HFD-fed mice. (**B1**): Representative recordings of mEPSCs in liver-related PVN neurons of control (upper trace) and HFD-fed (lower trace) mice. (**B2**,**B3**): Bar graphs illustrate that the frequency of mEPSCs (**B2**) was decreased in HFD-fed mice, whereas the amplitude of mEPSCs was similar (**B3**). (**C1**): Representative traces of mIPSCs in liver-related PVN neurons from control (upper trace) and HFD-fed (lower trace) mice. There was no difference in the frequency (**C2**) and amplitude (**C3**) of mIPSCs. HFD: high-fed diet, mEPSCs: miniature excitatory post-synaptic currents, mIPSCs: miniature inhibitory post-synaptic currents. Circles and squares represent individual neurons. * *p* < 0.05.

To determine the excitability of liver-related PVN neurons, after a hyperpolarizing step, depolarizing current steps were applied, and the current–action potential frequency responses were compared in mice fed with control and HFD. The firing rate of liver-related PVN neurons was higher in HFD-fed mice compared with neurons from control mice (simple linear regression, HFD: R^2^ = 0.59, Y-intercept = 5.08 Hz, *n* = 9 from 8 mice vs. control: R^2^ = 0.50, Y-intercept = 3.22 Hz, *n* = 10 from 8 mice; *p* = 0.0391) (Figure 1A). There was no difference in slope (control: 0.39 Hz/pA vs. HFD: 0.40 Hz/pA, *p* > 0.05), indicating that the difference in firing was maintained during the entire current step protocol (0–30 pA). These data suggest that HFD increases the excitability of liver-related PVN neurons.

Next, we determined the excitatory and inhibitory synaptic regulation of liver-related PVN neurons. In HFD-fed mice, the average mEPSC frequency was lower than in control mice (HFD: 0.48 ± 0.10 Hz (ranged from 0.21 to 1.11 Hz; *n* = 9 from 6 mice) vs. control: 1.32 ± 0.38 Hz (ranged from 0.47 to 3.00 Hz), *n* = 7 from 5 mice) (unpaired *t*-test *p* = 0.0313) (Figure 1(B1,B2)). The amplitude of mEPSCs was not different between control and HFD-fed mice (control: 14.63 ± 1.40 pA (ranged from 10.71 to 20.06 pA), *n* = 7 from 5 mice vs. HFD: 12.13 ± 0.57 pA (ranged from 9.76 to 14.93 pA), *n* = 9 from 6 mice) (unpaired *t*-test, *p* > 0.05) (Figure 1(B3)).

Recordings of mIPSCs did not show a difference between control and HFD-fed mice regarding frequency (control: 1.73 ± 0.60 Hz (ranged from 0.18 to 4.06 Hz), *n* = 7 from 7 mice vs. HFD: 0.77 ± 0.46 Hz (ranged from 0.13 to 3.01 Hz), *n* = 6 from 4 mice) (Mann–Whitney test *p* > 0.05) or amplitude (control: 19.79 ± 3.26 pA (ranged from 10.92 to 34.35 pA), *n* = 7 from 7 mice vs. HFD: 13.52 ± 3.55 pA (ranged from 8.12 to 22.54 pA), *n* = 6 from 4 mice) (Mann–Whitney test *p* > 0.05) (Figure 1C).

### 3.2. The Excitability of Liver-Related PVN Neurons Is Decreased in Response to Insulin in Mice Fed with HFD

The effect of insulin was assessed on the resting membrane potential, firing activity, and synaptic regulation of liver-related PVN neurons. At baseline, in control mice, bath application of insulin did not have a significant effect on resting membrane potential (−47.77 ± 1.61 mV (ranged from −59.00 to −40.77 mV) vs. −50.12 ± 2.08 mV (ranged from −63.84 to −40.75 mV), *n* = 12 from 9 mice) (paired *t*-test *p* > 0.05) or baseline firing activity (0.79 ± 0.37 Hz (ranged from 0.01 to 3.32 Hz) vs. 0.51 ± 0.26 Hz (ranged from 0.00 to 2.22 Hz), *n* = 8 from 7 mice]) (Wilcoxon test *p* > 0.05) (Supplementary Figure S1(B1,B2)). In contrast, in HFD-fed mice, insulin application decreased the baseline firing activity of liver-related PVN neurons from 0.83 ± 0.28 Hz (ranged from 0.04 to 2.28 Hz, *n* = 10 from 8 mice) to 0.47 ± 0.22 Hz (ranged from 0.00 to 1.75 Hz, *n* = 10 from 8 mice) (Wilcoxon test *p* = 0.0117), while the resting membrane potential was unaltered (−45.08 ± 1.52 mV vs. −46.40 ± 1.61 mV, *n* = 12 from 9 mice) (paired *t*-test *p* > 0.05) (Supplementary Figure S1(C1,C2)).

**Figure 2:**
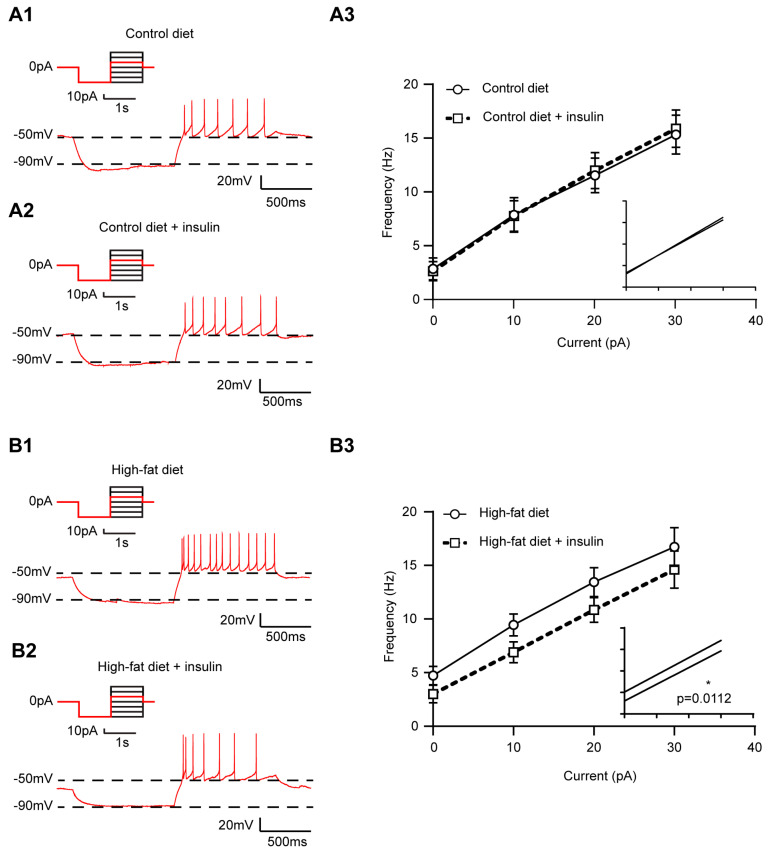
In HFD-fed mice, the firing of liver-related PVN neurons was reduced in response to insulin. (**A**): Representative traces illustrate the firing of liver-related PVN neurons before (**A1**) and after (**A2**) insulin application in control mice. The recorded neurons were initially hyperpolarized to ~−90 mV, then depolarizing current steps were applied to reveal the firing activity before and after insulin. Multiple current steps were applied; however, only one trace (+10 pA) is shown for better visibility. (**A3**): In control mice, the current–action potential frequency relationship demonstrated that the firing of liver-related PVN neurons did not change in response to insulin. (**B**): Representative recordings illustrate the firing rate of a liver-related PVN neuron before (**B1**) and after (**B2**) bath application of insulin. (**B3**): Current–action potential frequency responses to insulin and computed simple linear regression in HFD-fed mice demonstrate that liver-related PVN neurons fired less. HFD: high-fed diet, mEPSCs: miniature excitatory post-synaptic currents, mIPSCs: miniature inhibitory post-synaptic currents. * *p* < 0.05.

The action potential frequency following current injections was consistent with these findings. In control mice, insulin did not change the excitability of liver-related PVN neurons (simple linear regression, before insulin: R^2^ = 0.53, Y-intercept = 3.32 Hz vs. after insulin: R^2^ = 0.58, Y-intercept = 3.06 Hz, *n* = 9 from 8 mice, *p* > 0.05) or the slope (before insulin: 0.41 Hz/pA vs. after insulin: 0.44 Hz/pA, *p* > 0.05) (Figure 2A). In contrast, in HFD-fed mice, the simple linear regression of current–action potential frequency revealed a reduction in firing rate after insulin application (before insulin: R^2^ = 0.59, Y-intercept = 5.08 Hz vs. after insulin: R^2^ = 0.61, Y-intercept = 3.02 Hz, *n* = 9 from 8 mice) (*p* = 0.0112), whereas the slope was comparable (before insulin: 0.40 Hz/pA vs. after insulin: 0.39 Hz/pA, *p* > 0.05) (Figure 2B). These data suggest that the excitability of liver-related PVN neurons is decreased in response to insulin in HFD-fed mice.

**Figure 3:**
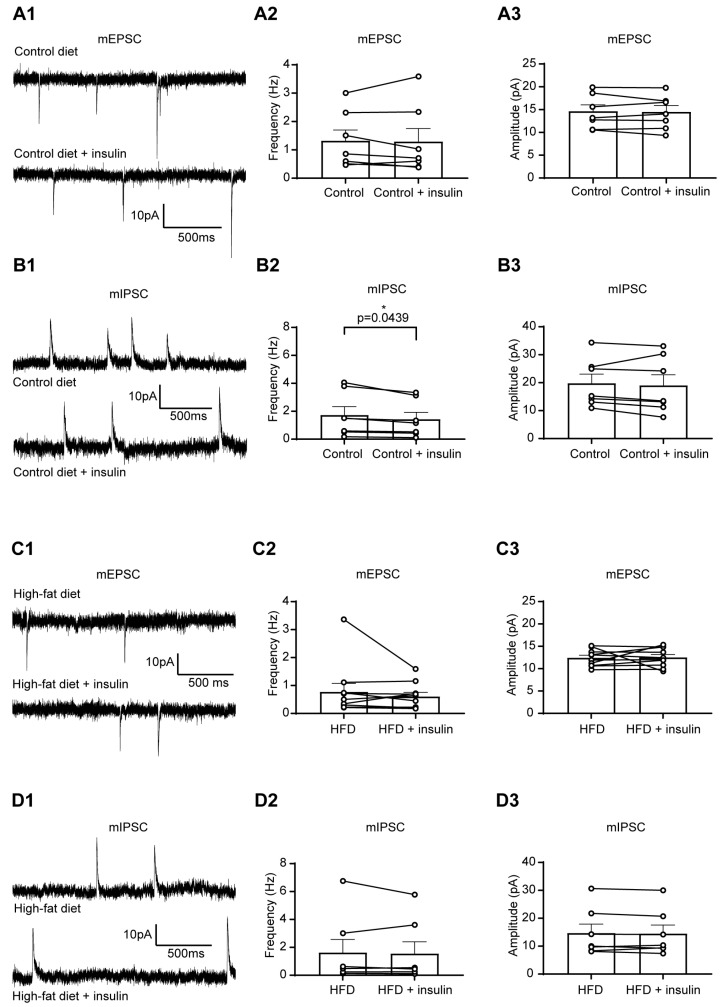
The insulin-dependent suppression of inhibitory neurotransmission is diminished in HFD-fed mice. (**A**,**B**): Representative traces illustrate the effect of insulin on mEPSCs (**A1**) and mIPSCs (**B1**) in control mice. In control mice, insulin did not alter the frequency (**A2**) and amplitude (**A3**) of mEPSCs; however, it decreased the frequency (**B2**) of mIPSCs without altering the amplitude (**B3**). (**C**,**D**): Representative traces illustrate the effect of insulin on mEPSCs (**C1**) and mIPSCs (**D1**) in HFD-fed mice. In HFD-fed mice, insulin did not alter the frequency and amplitude of mEPSCs (**C2**,**C3**) and mIPSCs (**D2**,**D3**). HFD: high-fed diet, mEPSCs: miniature excitatory post-synaptic currents, mIPSCs: miniature inhibitory post-synaptic currents. Circles and squares represent individual neurons. * *p* < 0.05.

Insulin application did not lead to a significant change in the frequency of mEPSCs of liver-related PVN neurons neither in control (before insulin: 1.32 ± 0.38 Hz vs. after insulin: 1.29 ± 0.46 Hz, *n* = 7 from 5, paired *t*-test *p* > 0.05) (Figure 3(A1,A2)) nor in HFD-fed mice (before insulin: 0.77 ± 0.30 Hz vs. after insulin: 0.61 ± 0.15 Hz, *n* = 10 from 5 mice, Wilcoxon test *p* > 0.05) (Figure 3(C1,C2)). Similarly, there was no difference in amplitude of mEPSCs before or after insulin application (control: 14.63 ± 1.40 pA vs. 14.48 ± 1.39 pA, *n* = 7 from 5 mice, paired *t*-test *p* > 0.05; HFD: 12.42 ± 0.59 pA vs. 12.51 ± 0.67 pA, *n* = 10 from 5 mice, paired *t*-test *p* > 0.05) (Figure 3(A3,C3)).

On the other hand, insulin application caused a decrease of mIPSC frequency in liver-related PVN neurons of control mice from 1.73 ± 0.60 Hz (ranged from 0.18 to 4.06 Hz) to 1.43 ± 0.49 Hz (ranged from 0.11 to 3.34 Hz, *n* = 7 from 6 mice) (paired *t*-test *p* = 0.0439) (Figure 3(B1,B2)); however, this insulin-induced reduction of mIPSC frequency was absent in liver-related PVN neurons of HFD-fed mice (before insulin: 1.63 ± 0.94 Hz (ranged from 0.13 to 6.76 Hz) vs. after insulin: 1.55 ± 0.85 Hz (ranged from 0.10 to 5.78 Hz), *n* = 7 from 6 mice) (Wilcoxon test *p* > 0.05) (Figure 3(D1,D2)). The amplitude of mIPSCs was not affected by insulin in control or HFD-fed mice (control mice: 19.79 ± 3.26 pA vs. 19.01 ± 3.80 pA, *n* = 7 from 6 mice and HFD-fed mice: 14.70 ± 3.22 pA vs. 14.48 ± 3.09 pA, *n* = 7 from 6 mice) (paired *t*-test *p* > 0.05) (Figure 3(B3,D3)).

### 3.3. HFD and Insulin Do Not Alter the Excitability of VLM-Projecting Liver-Related PVN Neurons

**Figure 4:**
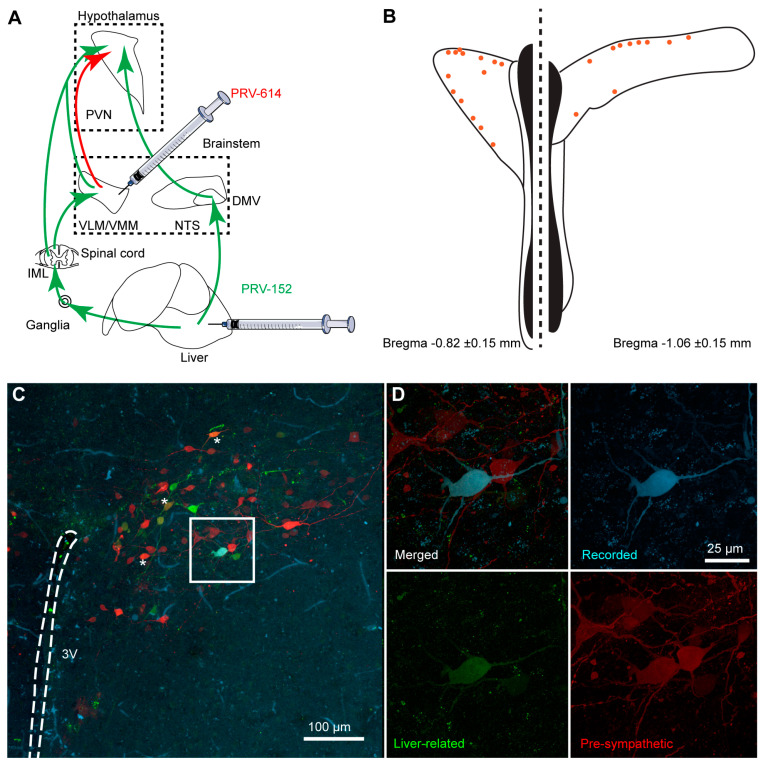
Identification of liver-related neurons in the PVN with projections to the ventral brainstem. (**A**): Schematic illustration of the viral inoculations. The liver was inoculated with PRV-152 (green) to identify liver-related neurons, then PRV-614 (red) was injected into the VLM to identify PVN neurons with projections to the ventral brainstem. Double-labeled neurons represent VLM-projecting liver-related PVN neurons. (**B**): Location of recorded pre-sympathetic, VLM-projecting liver-related PVN neurons. Orange dots represent the approximate location of the recorded neurons. The dotted line represents the 3^rd^ ventricle. Note the different distance from Bregma (left and right), representing two distinct parts of the PVN. (**C**): A confocal image (20×) of the PVN illustrates liver-related neurons (green), VLM-projecting neurons (red), and liver-related neurons with projections to the VLM (yellow/orange). The stars indicate some of the ventral brainstem projecting liver-related neurons. Dotted line indicates the 3^rd^ ventricle. Boxed area is enlarged on (**D**). (**D**): Enlarged image of the boxed area on C illustrates a recorded neuron (blue, 60×), which is a liver-related VLM-projecting neuron in the PVN. 3V: third ventricle; DMV: dorsal motor nucleus of the vagus; IML: intermediolateral nucleus of the spinal cord; NTS: nucleus tractus solitarius; PRV: pseudorabies virus; PVN: paraventricular nucleus of the hypothalamus; VLM: ventrolateral medulla.

In these experiments, we determined the excitability of liver-related PVN neurons with projections to the VLM. PRV-614 (RFP) was injected into the VLM in addition to the inoculation of the liver with PRV-152 (GFP); therefore, the double-labeling identified pre-sympathetic, VLM-projecting, liver-related PVN neurons. Figure 4 illustrates the experimental design that allowed the identification of liver-related PVN neurons based on their projection.

Unexpectedly, the baseline properties of VLM-projecting liver-related PVN neurons were similar in mice kept on control or HFD. We did not find a difference in the resting membrane potential, baseline firing activity, or synaptic neurotransmission (Table 1).

**Figure 5:**
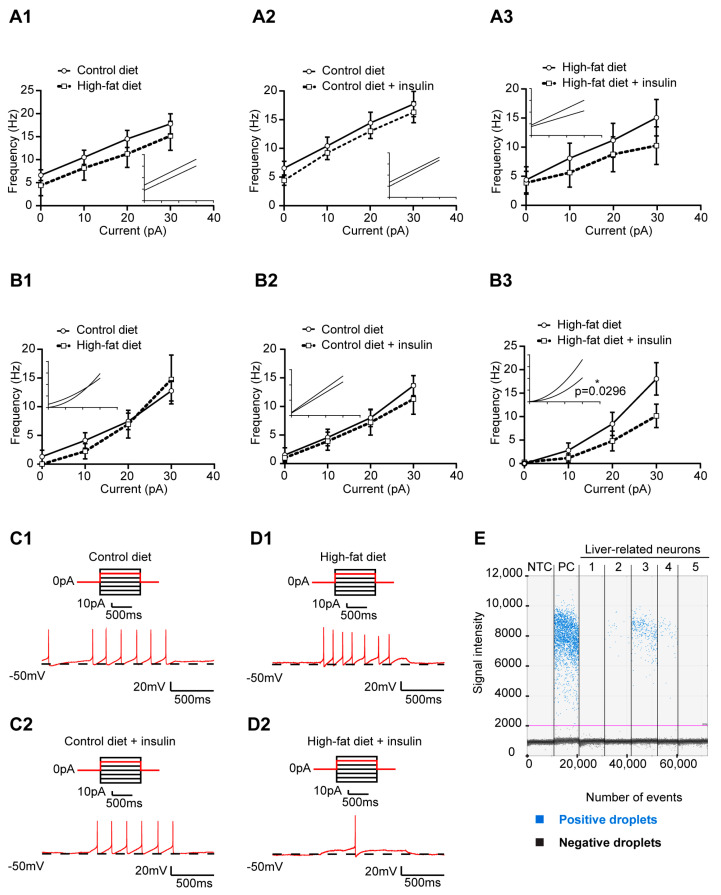
The excitability of liver-related neurons in the VLM/VMM decreased in response to insulin in mice fed with HFD. (**A**): Current–action potential frequency relationship demonstrates that diet (**A1**) or insulin (**A2**,**A3**) did not have an effect on the excitability of VLM-projecting liver-related PVN neurons. (**B1**): Diet did not affect the firing rate of pre-sympathetic liver-related neurons in the VLM/VMM. (**B2**,**B3**): Whereas in control mice, insulin did not alter the firing, it suppressed the excitability of liver-related neurons in the VLM/VMM in HFD-fed mice (**B3**). (**C**)**:** Representative traces illustrate the firing rate of liver-related VLM/VMM neurons before (**C1**) and after (**C2**) bath application of insulin in control mice.(**D**): Representative traces illustrate the firing rate of liver-related VLM/VMM neurons before (**D1**) and after (**D2**) bath application of insulin in HFD-fed mice. Whereas multiple current steps were applied, only one trace (+10 pA) is shown for better visibility. (**E**): Using droplet digital PCR insulin receptor expression (blue dots) was shown in a subset of liver-related PVN neurons. NTC: non-template control; PC: positive control. * *p* < 0.05.

The action potential frequency following current injection was not significantly different in VLM-projecting liver-related PVN neurons in control and HFD-fed mice (control: R^2^ = 0.49, Y-intercept = 6.74 Hz, *n* = 7 from 6 mice vs. HFD: R^2^ = 0.29, Y-intercept = 4.45 Hz, *n* = 6 from 5 mice, *p* > 0.05). There was no difference in slope (slope = 0.37 Hz/pA vs. slope = 0.35 Hz/pA Hz/pA, *p* > 0.05) (Figure 5(A1)).

Then, the effect of insulin was determined on liver-related PVN neurons projecting to the VLM. Insulin did not affect the membrane properties or the frequency of synaptic currents in control or HFD-fed mice (Table 1). Similarly, insulin application did not result in a significant change of the firing rate of VLM-projecting liver-related PVN neurons in response to current injection neither in control or HFD-fed mice (simple linear regression, control: R^2^ = 0.49, Y-intercept = 6.74 Hz, slope = 0.37 Hz/pA vs. R^2^ = 0.64, Y-intercept = 4.90 Hz, slope = 0.39 Hz/pA, *n* = 7 from 6 mice, *p* > 0.05 and HFD: R^2^ = 0.29, Y-intercept = 4.45 Hz, slope = 0.35 Hz/pA vs. R^2^ = 0.14, Y-intercept = 3.82 Hz, slope = 0.22 Hz/pA, *n* = 6 from 5 mice, *p* > 0.05) (Figure 5(A2,A3)).

### 3.4. Insulin Decreases the Excitability of Liver-Related Neurons in the VLM/VMM in HFD-Fed Mice

Since sympathetic discharge is largely modulated by the activity of pre-sympathetic neurons in the VLM/VMM, we tested the hypothesis that HFD alters the excitability of liver-related neurons at the level of the brainstem. Pre-sympathetic neurons were recorded 96 h after inoculation of the liver in control and HFD-fed mice. At baseline, the membrane properties and firing rate of liver-related neurons were similar in control and HFD-fed mice (control: −48.49 ± 2.00 mV, *n* = 17 from 9 mice, 3.32 ± 1.24 Hz, *n* = 8, from 6 mice vs. HFD: −49.57 ± 2.19 mV, *n* = 11 from 5 mice, 3.71 ± 1.55 Hz, *n* = 5 from 5 mice, unpaired *t*-test, *p* > 0.05) (Supplementary Figure S1(D1,D2)). The action potential frequency in response to current injections did not show a difference in the excitability of liver-related VLM/VMM neurons between control and HFD-fed mice (non-linear fit, similar curves in control (R^2^ = 0.56, Sy.x = 3.98), *n* = 8 from 6 mice and HFD-fed mice (R^2^ = 0.55, Sy.x = 5.51), *n* = 5 from 3 mice, *p* > 0.05) (Figure 5(B1)).

Then, the effect of insulin on the firing of liver-related VLM/VMM neurons was determined. In mice kept on control diet, insulin did not affect the firing rate of neurons following current injection (simple linear regression, before insulin: R^2^ = 0.58, Y-intercept = 0.95 Hz, slope = 0.40 Hz/pA vs. after insulin: R^2^ = 0.39, Y-intercept = 0.74 Hz, slope = 0.34 Hz/pA, *n* = 7 from 6 mice, *p* > 0.05) (Figure 5(B2,C1,C2)). In contrast, in HFD-fed mice, the firing rate of liver-related VLM/VMM neurons in response to current injection was reduced after insulin application (non-linear fit, different curves before and after insulin (R2 = 0.76, Sy.x= 4.36 vs. R2 = 0.63, Sy.x = 3.28), *n* = 4 from 2 mice, *p* = 0.0296) (Figure 5(B3,D1,D2)).

### 3.5. Insulin Receptor Expression in Liver-Related Neurons

Since our data show that insulin affects the excitability of liver-related neurons in the PVN and VLM/VMM, we verified insulin receptor expression in some of the recorded neurons. Using single-cell ddPCR, insulin receptor gene expression was detected in a population of liver-related PVN and VLM/VMM neurons (Figure 5E). In the PVN, in control mice, insulin receptor gene expression was detected in five out of eight liver-related neurons. Similarly, in HFD-fed mice, we were able to detect insulin receptor mRNA in four out of seven liver-related PVN neurons. We also verified insulin receptor expression in a population of pre-sympathetic liver-related VLM/VMM neurons (control: 13 out of 19 vs. HFD: 5 out of 14). These data confirm that a subset of liver-related neurons expresses insulin receptors.

## 4. Discussion

Our study revealed that feeding with HFD altered the excitability of neurons that are part of the brain–liver circuit. Our main findings are: (1) the excitability of liver-related PVN neurons is increased in HFD-fed mice, (2) the firing activity of liver-related PVN neurons is decreased in response to insulin in HFD-fed mice, (3) diet and insulin did not affect the firing of VLM-projecting liver-related PVN neurons, (4) the firing of liver-related neurons in the VLM/VMM is decreased in response to insulin in HFD-fed mice, and (5) a population of liver-related neurons expressed insulin receptors. The demonstration of increased activity of liver-related neurons further suggests altered central autonomic circuitry, likely contributing to increased sympathetic output and hepatic glucose production in diet-induced obesity.

### Plasticity of Liver-Related Neurons in HFD-Fed Mice

The sympathetic innervation of the liver originates from pre-ganglionic neurons in the intermediolateral column of the spinal cord (T7–T12), with projections to post-ganglionic neurons located mainly in the celiac and superior mesenteric ganglia [8,29,30]. The activity of sympathetic pre-ganglionic neurons is largely determined by inputs from neurons in supraspinal areas, including the VLM, VMM, and PVN [18]. The pre-sympathetic neurons are known to influence sympathetic outflow and glycemia. For instance, activation of PVN neurons—either with an excitatory neurotransmitter or by removing inhibition—resulted in elevated glucose levels due to activation of the SNS [12,31]. Similarly, at the level of the brainstem, stimulation of pre-sympathetic ventral brainstem neurons led to increased glucose levels and higher hepatic expression of PEPCK and G6Pase [31,32]. These studies strongly suggest that a PVN-VLM neuronal network is involved in the autonomic regulation of the liver, and activation of neurons within this pathway may lead to enhanced hepatic glucose production via activation of the SNS.

Male C57BL/6J DIO mice were fed with HFD to induce obesity and model early stages of type 2 diabetes mellitus [33], then recordings were conducted from liver-related neurons in the PVN. In our study, six-week-old mice were placed on control and HFD for 9–13 weeks, and their weight and non-fasting glucose levels were recorded before sacrifice. Mice kept on HFD were significantly heavier than mice on control diet; however, we did not observe a difference in non-fasting glucose levels. Consistent with previous reports, a recent study demonstrated that exposure to HFD leads to progressive weight gain, fasting hyperglycemia, and impaired glucose tolerance approximately 3 weeks after the start of HFD [33,34]. In addition, decreased insulin sensitivity was observed after 7 weeks of HFD, demonstrating that HFD-fed mice are glucose intolerant and insulin resistant. Although we did not measure fasting glucose levels or conduct glucose and insulin tolerance tests, it is reasonable to assume that the mice had impaired glucose metabolism because they were exposed to HFD for 9–13 weeks [33,34].

Our data demonstrate that liver-related PVN neurons are more active in HFD-fed mice, which suggests that diet affects neuronal excitability in the hypothalamus. This is consistent with previous studies showing increased basic firing rate of PVN neurons in obese Zucker rats [35]. In addition, overactivity of liver-related PVN neurons was also described in transgenic *db*/*db* mice [20], further indicating that the overall excitability of liver-related PVN neurons is increased in obese and/or diabetic conditions.

**Figure 6:**
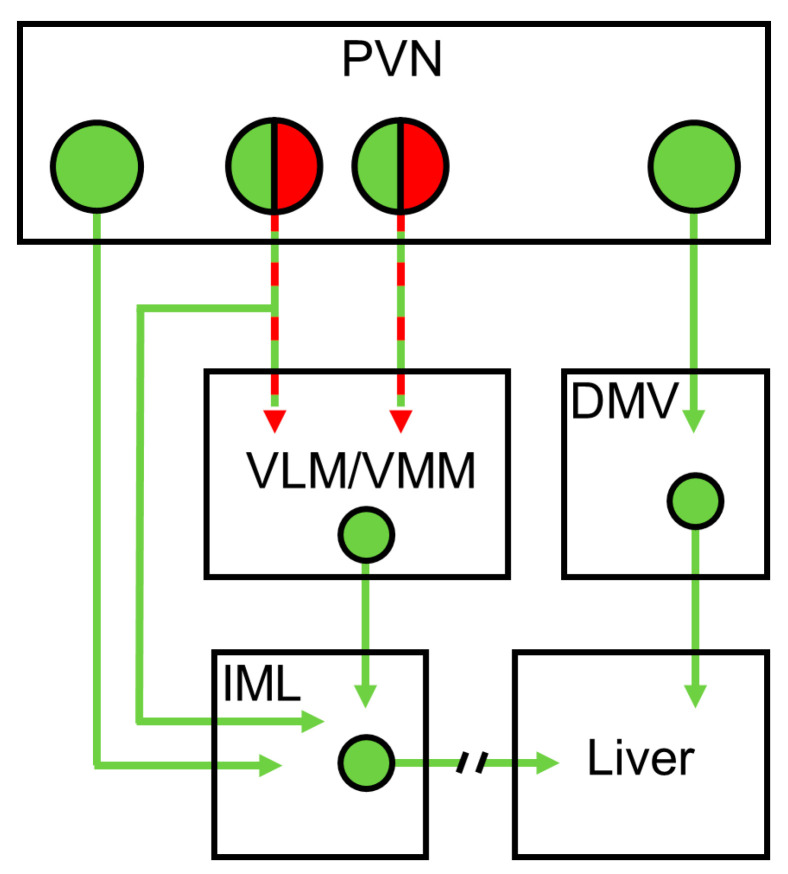
Schematic illustration of the PVN–liver pathway. Our data suggest that diet and insulin have differential effects on liver-related neurons depending on their location and projections. Green: liver-related neurons, green-red: liver-related PVN neurons with projections to the VLM/VMM. DMV: dorsal motor nucleus of the vagus, PVN: paraventricular nucleus, VLM/VMM: ventrolateral and ventromedial medulla.

In our study, liver-related neurons were visualized with PRV. While tracing with PRV allows the identification of liver-related neurons, this method does not distinguish pre-sympathetic and pre-parasympathetic neurons at the level of the PVN [11,19,20,36]. Increased activity of the SNS is associated with obesity; therefore, we identified a subset of pre-sympathetic neurons based on their projection to the ventral brainstem. Unexpectedly, the firing activity of VLM-projecting liver-related PVN neurons was not different in control and HFD-fed mice. These findings show that despite the general overactivity of liver-related PVN neurons in HFD-fed mice, the excitability of neurons with projections to the VLM did not change. These data suggest that either pre-sympathetic neurons with projections to the spinal cord and/or pre-parasympathetic neurons are behind the overactivity of liver-related PVN neurons in HFD mice (Figure 6). This would be consistent with observations from obese Zucker rats showing that the firing activity of PVN neurons with projections to the spinal cord is increased [35]; however, further studies are needed to confirm it.

Insulin resistance is one of the hallmarks of metabolic diseases, thus we revealed the activity of liver-related PVN neurons in response to insulin. Our data showed that in control mice, insulin did not change the overall firing activity of liver-related PVN neurons, whereas, in HFD-fed mice, bath application of insulin resulted in a decreased firing of liver-related PVN neurons. Since the overall activity of VLM-projecting PVN neurons did not change in response to insulin in control or HFD-fed mice, we investigated pre-sympathetic liver-related neurons in the ventral brainstem. We found that insulin application resulted in a reduction of firing activity in liver-related VLM/VMM neurons of HFD-fed mice, similar to what was observed in liver-related PVN neurons.

Previously, insulin was shown to directly inhibit a population of arcuate, ventromedial hypothalamus (VMH) and retrochiasmatic area neurons, while another population of neurons did not respond or depolarize after insulin application [37,38]. These studies also identified that the neurons responding to insulin were glucose-responsive neurons. Our data indicate that insulin does not affect the overall firing of liver-related neurons; however, we have to note that liver-related neurons had heterogeneous responses to insulin. In control mice, some of the recorded liver-related PVN neurons showed hyperpolarization (5/12), while others did not respond (5/12) or depolarize (2/12) following insulin application. This is consistent with a previous finding demonstrating that intracerebroventricular injection of insulin directly activates oxytocin-expressing PVN neurons measured by c-fos and changes in intracellular Ca^2+^ levels [39], and with the above-mentioned studies [37,38]. In addition, our ddPCR data demonstrate that a population of liver-related neurons contains insulin receptors, which is aligned with previous findings showing insulin receptor substrate immunopositivity in liver-related neurons [16]. Astrocytic insulin signaling plays an important role in the regulation of neurons [40], and PVN astrocytes control glucose metabolism and energy homeostasis [41]. In our study, we cannot exclude the potential influence of glial cells on the cellular properties of liver-related neurons, which is a limitation of our investigation. Nevertheless, central insulin actions are key processes in modulating peripheral glucose homeostasis, including hepatic glucose production [40,41,42,43], and central insulin resistance contributes to increased hepatic glucose production [44]. Ultimately, the PVN is very heterogenous and contains a variety of neurons; therefore, it is possible that only a specific subset of neurons, for instance, based on their phenotype or projection, will respond to insulin. However, identifying which subset of liver-related neurons is responsive to insulin remains to be determined.

Intriguingly, our data demonstrated that following a HFD, the excitability of liver-related neurons in the PVN and VLM/VMM is suppressed after insulin application. This is in contrast to earlier findings showing that insulin had no effect on membrane properties of glucose-responsive neurons in obese Zucker rats [38]. On the other hand, it is also possible that overactivated insulin signaling due to HFD will alter the sensitivity of neurons to insulin, as shown previously in mice with insulin receptor deficiency in SF-1 VMH neurons [45]. In these mice, on control diet, insulin-dependent silencing was absent; however, when insulin levels rose due to HFD, insulin action reached a threshold to activate the PI3K-K_ATP_ pathway leading to the decreased firing of POMC neurons [37,45]. In general, the intrinsic properties and the inhibitory and excitatory synaptic inputs determine the activity of neurons. In our studies, we found that the excitatory neurotransmission to liver-related PVN neurons was reduced in HFD-fed mice. Synaptic plasticity is known after dietary changes [46,47,48], including impairment of synaptic neurotransmission in HFD-fed rodents. In mice, an increased frequency of mEPSCs was observed in the VMH, decreased amplitude of mEPSCs in the arcuate nucleus and VMH, reduced frequency of mIPSCs in the lateral hypothalamus, and increased IPSCs amplitude in the PVN [47]. Moreover, time-dependent synaptic plasticity induced by HFD was observed in the lateral hypothalamus [49]. We also found that insulin-dependent suppression of inhibitory neurotransmission in liver-related neurons was diminished in HFD-fed mice, which is aligned with previous reports that HFD alters GABAergic neurotransmission in the dorsomedial hypothalamus [50]. In summary, our data extend these findings by demonstrating that HFD alters synaptic neurotransmission in liver-related neurons, which further suggests synaptic plasticity following dietary challenges.

## 5. Conclusions

Our data further support that HFD alters neuronal activity, in particular, the excitability of liver-related neurons and their response to insulin. The sympathetic nervous system largely contributes to the regulation of liver carbohydrate and lipid metabolism, and increased sympathetic activity is known in obesity and type 2 diabetes. The excitability of neurons determines the activity of sympathetic nerves; therefore, our study provides novel information about the mechanisms underlying the altered brain-liver circuit in obese mice.

## Figures and Tables

**Table 1:** Resting membrane potential, firing rate, and synaptic neurotransmission in VLM-projecting liver-related PVN neurons.

	Membrane Properties												mEPSC								mIPSC							
	RMP (mV)				AP Frequency (Hz)				Rin (GΩ)				Frequency (Hz)				Amplitude (pA)				Frequency (Hz)				Amplitude (pA)			
	Mean	SEM	n	*p*	Mean	SEM	n	*p*	Mean	SEM	n	*p*	Mean	SEM	n	*p*	Mean	SEM	n	*p*	Mean	SEM	n	*p*	Mean	SEM	n	*p*
Control diet	−50.71	2.17	10	0.69	0.79	0.40	6	0.61	1.00	0.09	10	0.38	1.17	0.17	9	0.29	14.47	1.19	9	0.78	0.81	0.16	11	0.14	13.43	0.70	11	0.07
High-fat diet	−49.43	2.30	8		1.20	0.52	4		1.15	0.16	8		1.50	0.22	14		14.90	0.99	14		1.21	0.19	15		17.10	1.55	15	
Control diet	−50.71	2.17	10	0.27	0.59	0.32	8	0.25	1.00	0.09	10	0.21	1.62	0.40	8	0.06	13.77	1.50	8	0.66	0.79	0.18	10	0.60	13.17	0.72	10	0.20
Control diet + Insulin	−49.35	1.81	10		0.25	0.06	8		1.07	0.11	10		1.40	0.37	8		14.06	1.74	8		0.86	0.15	10		12.08	0.74	10	
High-fat diet	−49.43	2.30	8	0.68	1.20	0.52	4	>0.99	1.151	0.16	8	0.54	1.54	0.24	12	0.30	14.59	0.87	12	0.08	1.27	0.23	11	0.08	18.02	1.93	11	0.01
High-fat diet + Insulin	−48.87	2.91	8		1.09	0.49	4		1.07	0.16	8		1.50	0.44	12		13.32	1.12	12		1.13	0.22	11		16.17	1.56	11	

## Data Availability

The data presented in this study are available on request from the corresponding author.

## Supplementary Materials

The following supporting information can be downloaded at: https://www.mdpi.com/article/10.3390/cells12081194/s1, Figure S1: The effect of high-fat diet and insulin on the cellular properties of liver-related neurons.
