# Supplementary material for: High-Fat Diet Modulates the Excitability of Neurons within the Brain–Liver Pathway
Source: Cells. 2023 Apr 20;12(8):1194. doi: 10.3390/cells12081194 (PMC10137256; doi:10.3390/cells12081194)
Supplement: Supplementary file 1 [file cells-12-01194-s001.zip › cells-2320367-supplementary.pdf]

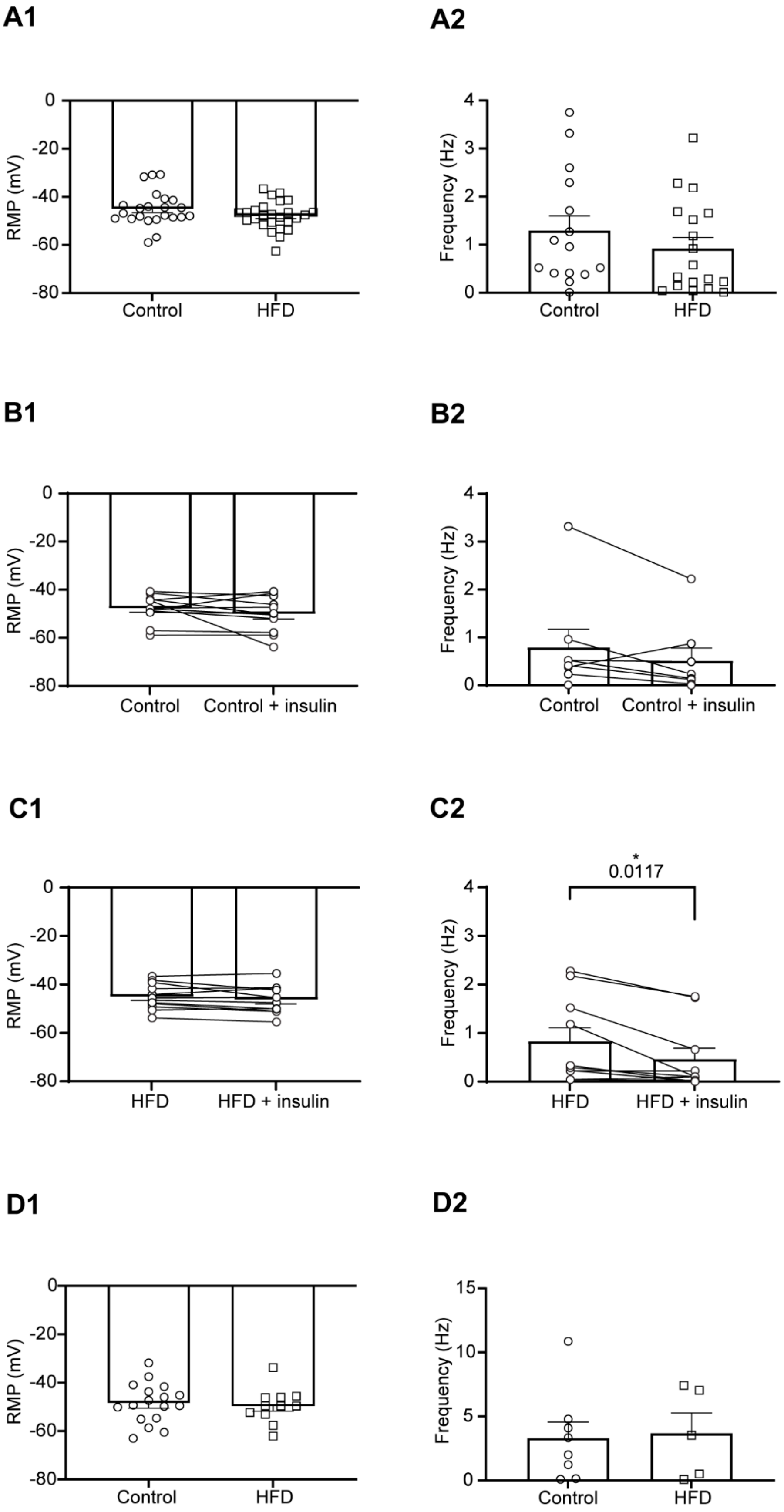

Figure S1:The effect of high-fat diet and insulin on the cellular properties of liver-related neurons.
